# Orthodontic Approaches in the Management of Mandibular Fractures: A Scoping Review

**DOI:** 10.3390/children10030605

**Published:** 2023-03-22

**Authors:** Alessandro Polizzi, Vincenzo Ronsivalle, Antonino Lo Giudice, Gaetano Isola, Alberto Bianchi, Simona Santonocito, Rosalia Leonardi, Stefano Mummolo

**Affiliations:** 1 Department of General Surgery and Surgical-Medical Specialties, School of Dentistry, University of Catania, 95124 Catania, Italysimonasantonocito.93@gmail.com (S.S.);; 2 Department of Life, Health & Environmental Sciences, Postgraduate School of Orthodontics, University of L’Aquila, 67100 L’Aquila, Italy; 3 Department of General Surgery and Medical Surgery Specialties, Section of Maxillofacial Surgery, University of Catania, 95100 Catania, Italy

**Keywords:** mandibular fracture, children, direct bonding technique, orthodontic bracket, occlusal splint, elastics

## Abstract

Non-surgical approaches have been proposed in the management of mandibular fractures, especially in children, but there is a lack of clear guidelines on the clinical indications of conservative approaches. The aim of this scoping review is to provide the available evidence of the role of the orthodontist in the management of mandibular fractures. The PRISMA-ScR guidelines were followed to select eligible articles from the PubMed, Scopus, and Web of Science databases according to precise inclusion criteria. The research questions were formulated as follows: “what is the scientific evidence concerning the rule of orthodontists in the management of mandibular fractures” and “the preferential use of the direct bonding technique with orthodontic brackets rather than rigid arch bars”? Seventeen articles were included. Five articles presented the use of removable acrylic splints or functional appliances, six articles concerned the employment of cemented acrylic or rigid splints, and six articles described the management of mandibular fractures in adults and children using orthodontic brackets or mini-screws. Most of these techniques have been employed in children and growing subjects, while fewer data were available regarding conservative treatments in adults. Preliminary evidence suggests that condylar and some minor parasymphyseal fractures in children may be managed with conservative approaches. In adults, minor condylar and stable body mandibular fractures with minimal displacement have been reduced similarly. However, there are no sufficient elements that could suggest the preferential use of orthodontic brackets over rigid arch bars in adults. Further randomized and non-randomized clinical trials with long follow-ups will be needed to better define the clinical indications of the orthodontic approaches in the management of mandibular fractures based on severity, location, and age.

## 1. Introduction

The mandible is a U-shaped bone that connects to the cranial base at the temporomandibular joints and functions as an interface with the maxilla through the oral occlusion [1,2,3,4,5,6]. In total, 10 to 25 percent of all facial injuries are mandibular fractures, with interpersonal attacks being the main reason for these fractures, although several cases of facial trauma caused by the use of electric scooters have been reported recently [7,8,9,10,11,12,13].

The mechanism of the injury is crucial to evaluate since it gives the doctor a hint as to whether there are any concurrent injuries that can impede healing or call for a different type of therapy [1,14,15,16,17,18,19]. The condition of the occlusion, which can be aberrant in more than 80% of mandibular fractures, is the most crucial element in determining whether a mandible fracture needs a surgical intervention [20,21]. It is crucial to identify the type of occlusion that was present before the trauma because, even if the preinjury occlusion was atypical, the ultimate goal of any surgical intervention is to restore it [1,21,22].

Open reduction internal fixation (ORIF) has emerged as the gold standard of treatment for achieving anatomic reduction for a range of mandibular fractures, including condylar head fractures, despite the therapeutic value of a closed reduction of mandible fractures with mandibulomaxillary fixation (MMF) [23,24]. ORIF allows a fracture to be reduced in an efficient and safe way and may be implemented even in cases where a non-surgical approach failed [25,26]. However, in contrast to MMF, mandible ORIF is thought to carry a higher risk of postoperative infection problems [27,28,29,30]. The previous arch form and facial width may not be sufficiently restored by fractured bone reduction, which causes inconsistencies between dental occlusion and bone fracture reduction [31]. In addition to open surgical approaches with titanium plates, there are also alternative closed fixation treatments that can be implemented in various ways, including Erich arch bars, bone-supported arch bars, a direct bonding technique with orthodontic brackets, and passive splinted archwires [32,33,34,35,36,37,38]. Depending on the nature and location of a fracture, several approaches may be recommended for treating jaw fractures [33,39,40,41]. The disadvantages of an open surgical approach or the use of arch bars include postoperative discomfort, difficulties in maintaining oral hygiene, periodontal damage, and unpleasant and stressful procedures [42,43]. After removing an arch bar, for example, harmful effects of enamel demineralization and gingival irritation may be observed. In this regard, there are cases in which a dental intermaxillary fixation may be chosen (with the eventual involvement of an orthodontist) [33], which is a less invasive and more conservative approach. However, there are no guidelines that clarify whether and when an orthodontist may really play a role in the clinical management of fracture reduction.

A separate discussion should occur regarding children and growing subjects. The occurrences of facial fractures reach a peak throughout puberty and adolescence due to increased sport activity [44,45,46,47,48,49]. When treating pediatric patients, the impact of a trauma or treatment on growth and development is of the utmost importance. Pediatric patients are difficult to handle, and management is quite difficult, especially in the stage of mixed dentition. Children require a different set of care guidelines for mandibular fractures. In most situations, a cautious approach is recommended. Restoring the underlying bone architecture to its preinjury position in a stable manner while minimizing any remaining cosmetic and functional impairments is the aim of treatment for these fractures [50]. With titanium plates and screws or absorbable plates and screws, the open reduction and osteosynthesis of pediatric fractures involve a risk of impairing skeletal growth and harming erupting teeth. Therefore, a closed reduction is typically recommended [49,51,52,53,54].

In this regard, the aim of this scoping review is to provide the available evidence of the role of the orthodontist in the management of mandibular fractures.

## 2. Materials and Methods

### 2.1. Searching Strategies

This scoping review followed the PRISMA-ScR guidelines [55]. Using a combination of MeSH terms and free text words pooled through Boolean operators (‘AND’ and ‘OR’) in the PubMed, Scopus, and Web of Science databases, a search strategy without a timeline setting was implemented on 1 February 2023 to find all articles related to orthodontic approaches in the management of mandibular fractures. The research questions were formulated as follows: “what is the scientific evidence concerning the rule of orthodontists in the management of mandibular fractures” and “the preferential use of the direct bonding technique with orthodontic brackets rather than rigid arch bars?”

### 2.2. Article Selection

In the present scoping review, the selection of articles was based on precise inclusion and exclusion criteria. In particular, articles in English, without timeline restrictions, were only included if they presented at least one conservative method of orthodontic mandibular fracture management. With regard to the study design, the following publications were taken into consideration: randomized and non-randomized controlled clinical trials, cohort studies, case-control studies, cross-sectional studies, retrospective studies, case reports, and case series. On the other hand, all articles not relevant to the orthodontic management of mandibular fractures, not available in English and with the full text, and some study designs (opinion articles, theses, conference reports, and any kind of review article) were excluded.

Two independent reviewers were involved in the article selection processes. After removing duplicates, papers were initially screened for titles and abstracts. The full-text versions of all articles satisfying the inclusion criteria were further analyzed. After comparing the final results, if any disagreement occurred a third reviewer was involved. The following information was taken from the chosen articles: the study and the year of publication, the aim, the sample (including age), the design of the study, the site of the fracture/s, the approach, and the main findings.

## 3. Results

### 3.1. Study Selection

**Figure 1:**
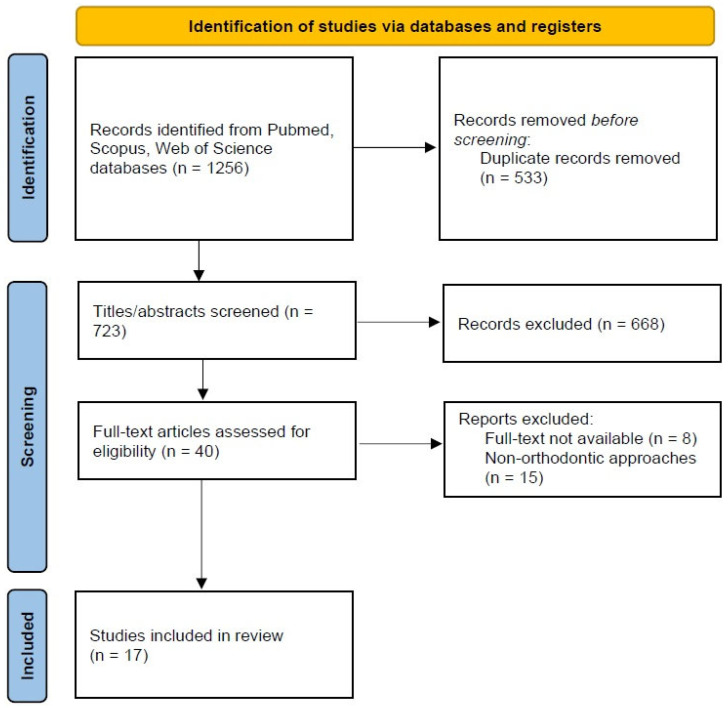
PRISMA-ScR flow diagram of the articles’ identification.

The search strategy led to the identification of a total of 1256 records in the PubMed (n = 552), Scopus (n = 497), and Web of Science (n = 207) databases (Table 1). Next, 553 duplicates were removed, and the remaining 723 articles were screened for titles and abstracts (Figure 1). Then, 668 records were excluded, and 40 papers were assessed for eligibility though a full-text analysis. After meticulous evaluation, 8 papers were excluded because full texts were not available, and 15 other papers were not included in this scoping review because they concerned the use of non-orthodontic approaches. Thus, at the end of the selection process, 17 articles were considered for this scoping review [56,57,58,59,60,61,62,63,64,65,66,67,68,69,70,71,72].

### 3.2. Description of the Included Studies

Table 2 presents the data extracted from the selected articles. The sizes of the analyzed samples ranged from 1 to 40 subjects, and the follow-up periods (when specified) ranged from 2 months to 10 years. Twelve studies [56,57,58,59,61,63,64,65,68,70,71,72] included growing subjects (age range: 3–16 years), while five articles [60,62,66,67,69] included adults (age range: 16–55 years).

Regarding the study designs, most of the articles were case reports [57,59,63,65,66,68,71] or case series [56,60]. Other articles were designed with prospective approaches (comparative [67,69] or non-comparative [58]) but with a lack of a systematic randomized (controlled and non-controlled) enrolment design. Finally, four studies featured retrospective designs [62,64,70,72].

### 3.3. Mandibular Fracture Management

**Figure 2:**
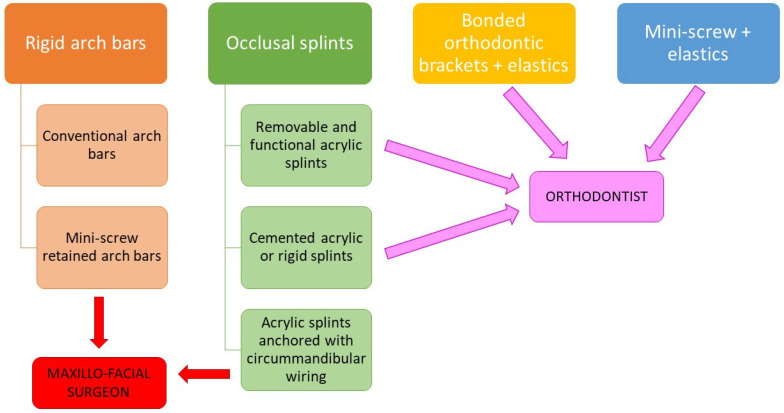
“Closed” approaches for the management of mandibular fractures.

In contrast to the intermaxillary fixation (IMF) approaches, which are usually managed by a maxillofacial surgeon, such as conventional or mini-screw-retained arch bars and occlusal splints fixed with circummandibular wiring, from the results of the present review, among the conservative IMF methods that are manageable by orthodontists and pediatric dentists, the following approaches emerged: (1) mini-screws and elastics [62,66,70]; (2) bonded orthodontic brackets and elastics [60,62,66,69,71]; (3) cemented [56,61,63,65,67] or (4) removable [59,68,72] occlusal splints; and (5) functional removable appliances [64], which may be associated with functional exercises (Figure 2). From the results of this review, these approaches have mainly been adopted for children and adolescents [56,57,58,59,61,63,64,65,68,70,71,72]. In some articles, these conservative approaches were presented and compared to the effects of acrylic splints fixed with circummandibular wires or arch bars [56,62,69] and the competence of the maxillofacial surgeons.

#### 3.3.1. Removable and Cemented Acrylic Splints

Many authors presented the use of acrylic splints that were removable [59,68,72] or cemented with glass ionomer cement [56,61,65] for the management of mandibular fractures. Sabbagh HT et al. [64] described the use of a functional appliance for the management of condylar fractures in growing subjects.

#### 3.3.2. Hybrid Protocols and Rigid Orthodontic Splints

Qadri GW et al. [63] employed a cemented acrylic splint associated with interdental wiring with 0.5 mm stainless-steel wire between the inferior incisors to support the reduction of a symphyseal fracture with minimal displacement. Instead, Aizendbud D et al. guided and achieved the reduction of a parasymphyseal fracture with minimal displacement using a rigid orthodontic splint consisting of two bands on primary second molars soldered with 1.2-inch rounded stainless-steel lingual and buccal archwires and short cross-archwires near the canines [57]. Trupthi DV et al. [67] employed a cemented vacuum-formed splint to guide the fracture reduction associated with the use of arch bars and elastics to maintain intermaxillary fixation, whereas Wu Y et al. [70] employed mini-screws for maxillomandibular fixation.

#### 3.3.3. Intermaxillary Fixation with Orthodontic Brackets and Mini-Screws

Other authors described fracture reduction with the use of fixed orthodontic appliances or brackets [60,62,66,69,71] and/or mini-screws with elastics [62,66] for intermaxillary fixation and the eventual use of acrylic splints to guide the reduction of fractures [58,62]. Functional exercises have also been successfully prescribed by some authors [58,59].

**Table 2:** Characteristics of the included studies.

Study	Aim	Sample	Design of the Study	Site of the Mandibular Fracture	Approach	Main Findings
Agarwal RM et al. (2014) [56]	To present a variety of therapeutic techniques for pediatric mandibular fractures.	19 subjects (3–12 y)	Case series	symphysis/parasymphysis (9), body (4), parasymphysis + condylar (4), body + condyle (1), and body + parasymphysis (1)	Acrylic cap splint (with circummandibular wiring or cemented) or Erich arch bar	Given the ages and anatomical diversity of children, managing pediatric mandibular fractures is difficult. Symphysis/parasymphysis fractures were the most prevalent kind of mandibular fracture in this case series, accounting for 42% of all fractures. Pediatric dentists play a crucial role in treating children who have such fractures. If the right guidelines are followed, children should obtain satisfactory results with the least amount of discomfort.
Aizendbud D et al. (2008) [57]	To describe the production of the orthodontic fixation device, treatment concerns, and long-term follow-up results (10 y).	1 subject (5 y)	Case report with 10 y follow-up	Parasymphysis with minimal displacement	Cemented rigid modified orthodontic splint appliance	Growth may be affected in an unforeseen way if the mandible’s periosteal envelope is compromised. Therefore, a closed reduction is preferred in children who need help. In this instance, the 10-year follow-up showed that the orthodontic fixation splint approach, which is noninvasive, can offer a superior and effective treatment for mandibular fracture in young children.
Boffano P et al. (2012) [58]	To present conservative measures as options for treating subjects with mixed dentition who had a series of unilateral displaced condylar fractures.	25 subjects (6–12 y)	Non-controlled prospective study with 1 y follow-up	Unilateral condylar with displacement	Fixed orthodontic appliances, acrylic splints, rubber bands, and functional exercises	At 12 months of follow-up, a conservative treatment of displaced unilateral condylar fractures in children with mixed dentition produced good functional results. The correct reconstruction of the condyles was guided by a splint that was progressively modified, allowing the fractured process to regain its normal forms and heights. Condylar fractures in kids between the ages of 6 and 13 can still be treated non-surgically, which is still the best option to preserve healthy growth and function.
Cazzolla AP et al. (2018) [59]	To present the therapeutic option of a condylar neck fracture in an 11-year-old boy.	1 subject (11 y)	Case report with 1 y follow-up	condylar neck with minimal displacement	Removable acrylic splint and functional exercises	With a lower-resin splint, a non-surgical treatment for a child’s condylar neck fracture can restore mandibular motions and appearance. After a year of treatment, facial growth was normal. In certain circumstances with a mildly displaced condyle, a conservative treatment may be recommended for children.
Chen CY et al. (2010) [60]	To propose an innovative method, the direct bonding technique, as an alternative to intermaxillary fixation with arch bars or looped wires.	2 subjects (19–28 y)	Case series with 3-month follow-up	Condylar or condylar + parasymphysis and body	Bonding of brackets and wires + elastics	This novel approach did not require the insertion of interdental wires in the typical manner. The removal of challenges, time requirements, and penetration risk thereby benefits the practitioner. Compared to arch bars or looped wires, it also lessens pain and tension for the patient, and dental hygiene is much improved.
Choubey S et al. (2014) [61]	To highlight the benefits of a vacuum-formed splint that was selected as a viable and successful conservative therapy strategy for the management of maxillofacial injuries.	1 subject (9 y)	Case report with 2 y follow-up	Parasymphysis with displacement	Cemented vacuum-formed splint	Compared to fractures in the adult population, pediatric maxillofacial traumas necessitate different therapeutic treatment approaches. The prognoses for many dentoalveolar injuries can be considerably improved with prompt and adequate care. The most crucial element, time, determines the course of treatment and its result.
Kocaaslan BK et al. (2022) [62]	To compare conservative treatment modalities for condylar fractures and determine the best option using CT images.	24 subjects (18–48 y)	Retrospective study with 6-month follow-up	condylar neck (bilateral or unilateral)	Bracketing, arch bar, or mini-screw (+ eventual occlusal splint)	No statistically significant differences were detected between the treatment modalities in the condylar length difference (affected side vs. unaffected side). An acceptable and more conservative form of treatment is intermaxillary fixation with an occlusal splint, as opposed to an open reduction.
Qadri GW et al. (2008) [63]	In order to reduce the risk of complications, a case of a juvenile mandibular fracture treated with direct interdental wire and an acrylic splint instead of absorbable plates and screws was discussed.	1 subject (14 y)	Case report with 6-month follow-up	Symphysis with minimal displacement	Interdental wiring (0.5 mm stainless-steel wire) + cemented acrylic splint	With the use of an acrylic splint and direct interdental wiring, the subject was successfully treated.
Sabbagh HT et al. (2022) [64]	To evaluate and monitor the progress of a conservative treatment strategy using a functional orthodontic appliance for the treatment of mandibular condyle fractures in young patients.	8 subjects (mean age: 8.3 y)	Retrospective study (follow-up not specified)	Condyle (bilateral or unilateral)	Functional removable appliance	The concept of a conservative functional approach in growing patients is supported by the positive functional and morphologic outcomes of functional orthodontic treatment. When treating growing patients with conservative measures for mandibular condyle fractures, functional adjunctive therapy should be taken into account.
Saskianti T et al. (2022) [65]	To aid pediatric dentists in managing this distinct and highly specialized area of traumatology by providing them with better knowledge on how to treat mandibular fractures in children.	1 subject (9 y)	Case report with 2-month follow-up	Symphysis/parasymphysis	Cemented closed-cap acrylic splint	Pediatric mandibular fractures treated with modified closed-cap splints, particularly those in the symphysis/parasymphysis region, are safe and successful.
Tehranchi A et al. (2013) [66]	To provide a case of a young subject who had maxillofacial trauma and needed cautious multidisciplinary care after suffering severe injuries in a plane crash.	1 subject (25 y)	Case report with 6-month follow-up	Symphysis and greenstick fracture at the anterior border of the ramus	Mini-screw and elastics + orthodontic brackets and wire	The proposed technique could constitute an adjunctive treatment to assist in the management of complex and multidisciplinary cases.
Trupthi DV et al. (2014) [67]	To assess the clinical effectiveness of arch bars against vacuum-produced splints in the management of mildly displaced mandibular fractures.	40 subjects (18–55 y)	Non-randomized comparative prospective study: group 1 (cemented vacuum-formed splint + elastics) vs. group 2 (arch bar), with 2-month follow-up	Minimally displaced mandibular fractures	Cemented vacuum-formed splint + elastics and arch bar	Regarding chair-side time, periodontal health, patient compliance with maintaining oral hygiene, mastication, and speech, vacuum-produced splints are superior to arch bars. Needlestick injuries are prevented by using vacuum-formed splints. Therefore, they can be applied to minimally displaced mandibular fractures for intermaxillary fixation.
Tuna EB et al. (2012) [68]	To clinically and radiologically review the results of the conservative approach of a 10-year-old subject who had a unilateral greenstick fracture for the previous 2.5 years.	1 subject (10 y)	Case report with 2.5 y follow-up	Unilateral greenstick condylar fracture	Removable acrylic mandibular splint	Children with unilateral fractures of the mandibular condyle may avoid deformation in future growth by using a non-surgical functional approach. Condylar growth could occur continuously and simultaneously during the growing period as a result of the proliferation in the chondrocyte layer, which promotes new bone formation in the fragmented condyle.
Utley DS et al. (1998) [69]	To evaluate the efficacy, ease of use, and safety profile of orthodontic direct bonded bracket fixation.	32 subjects (16–42 y)	Non-controlled prospective study with 44.4 ± 51.6-week follow-up (MMF/DDB or MMF/DDB + ORIF or arch bars)	Symphysis, angle, condylar neck, coronoid, and body.	Orthodontic brackets + elastics (with eventual ORIF) or arch bars	For less complicated subcondylar, angle, body, and symphyseal mandibular fractures, MMF/DBB is effective as the sole treatment strategy. In more difficult fractures, MMF/DBB is a useful preoperative addition to ORIF.
Wu Y et al. (2012) [70]	To assess the viability and safety of using an occlusal splint with a specially developed screw-based semi-rigid intermaxillary fixation in the conservative management of pediatric mandibular condylar fractures.	13 subjects (<12 y)	Retrospective study with 28.6-month mean follow-up	Condyle with displacement	Mini-screw and elastics + vacuum-formed occlusal splint	This approach may be a secure, simple, and efficient way to treat condylar fractures in children.
Xu YH et al. (2016) [71]	To describe a variant of the conservative fixed orthodontic treatment that was added to help a child with a bilateral condylar fracture heal.	1 subject (10 y)	Case report with 49-month follow-up	Condyle with displacement and symphysis	Bracket with 0.018 in Australian wire and elastics	A mandibular fracture can be treated conservatively with fixed orthodontics, which is an effective treatment approach that is generally simple, affordable, and acceptable to patients.
Zhao YM et al. (2014) [72]	To assess how a removable occlusal splint affects the treatment of condylar fractures in children and adolescents.	40 subjects (3–16 y)	Retrospective study with 14 months to 4 years of follow-up	Condyle	Removable occlusal splint	Condylar fractures in children can be successfully treated with conservative measures. A potential method for treating condylar fractures in children and teenagers is a removable occlusal splint.

MMF/DDB: orthodontic direct bonded bracket fixation; ORIF: open reduction/internal fixation; y: year/s.

## 4. Discussion

The aim of this scoping review is to give scientific evidence concerning the role of orthodontists in the management of mandibular fractures. The results of the present review show that conservative closed approaches that are manageable by orthodontists may be a good alternative in some types of mandibular fractures, especially in children and adolescents, while few studies have discussed the use of these methods in adults.

Most mandibular fractures need to be reduced and fixed in order to promote primary or secondary bone healing (if micromotions are allowed) with an intermediate callous healing phase. Intermaxillary fixation (IMF) with arch bars, occlusal splints, orthodontic brackets, or mini-screws may be used to execute this type of fixation. Instead, open reduction and internal fixation (ORIF) with plates and screws applied directly to the fracture site avoids the intermediate callous phase, prevents micromotions, and allows primary bone healing [1,73,74]. ORIF has the advantage of enabling direct viewing, quicker bone healing, and better nutrition and dental hygiene, but it is a more invasive procedure, causes discomfort to the patient, and can increase the risk of infectious and nervous complications [29]. On the other hand, IMF is less traumatic to the vascular envelope and for the patient, but a closed reduction involves a lengthy period of immobilization and closure of the oral cavity and necessitates an intact dentition or some kind of dental record; furthermore, it might not be the most advantageous choice in the long term [1,41].

The IMF approaches include the conventional [32,62,67,69] or mini-screw-retained arch bars [32,75,76] and occlusal splints fixed with circummandibular wiring that have also been applied in children and are usually managed by maxillofacial surgeons [56,77,78]. On the other hand, the most conservative orthodontic approaches may include the use of mini-screws and elastics [66], bonded orthodontic brackets and elastics [71], or removable or cemented occlusal splints with possible functional exercises [67,68].

### 4.1. Management of Mandibular Fractures in Adults

#### 4.1.1. Minimally Displaced Mandibular Body Fractures in Adults

A prospective comparative study [67] that enrolled adults (18–55 years) with minimally displaced mandibular fractures showed that the use of a cemented vacuum-formed splint + elastics had advantages in terms of chair-side time, periodontal health, patient compliance, oral hygiene, mastication, needlestick injuries, and speech compared to arch bars. The recommendation of conservative IMF devices in adults has been restricted for fractures with minimal displacement. Chen et al. [60] reported an adult subject with a displaced condylar fracture that was managed using ORIF and two stable parasymphyseal and body mandibular fractures with minimal displacement that were managed using orthodontic bonded brackets, archwires, and elastics. In this case, there were advantages of brackets as an alternative to arch bars or looped wires for the patient (less pain and better oral hygiene) and the dentist (interdental wiring was not required). Another adult subject with a symphyseal and greenstick fracture at the anterior border of the ramus [66] was successfully treated using orthodontic brackets and wires for the reduction of the mandibular fractures and using 10 mini-screws and light elastics to reinforce the anchorage of the IMF. Finally, a comparative prospective study [69] (direct bonding fixation vs. direct bonding fixation + ORIF or arch bars) concluded that for less complicated subcondylar, angle, body, and symphyseal mandibular fractures, a conservative orthodontic treatment could be considered as a single strategy, while for more complex fractures, ORIF remained the gold standard.

#### 4.1.2. Minor Condylar Fractures in Adults

The single patient with a condylar fracture in the case report of Chen et al. [60] was managed with bonded brackets and wires with elastics to assure IMF as an alternative to arch bars or looped wires to reduce patient discomfort and improve oral hygiene. Kocaaslan et al. [62] retrospectively compared the use of bonded brackets, arch bars, and mini-screws (with eventual acrylic splints to guide the reduction) in adult subjects for the management of condylar neck fractures. The authors did not detect significant differences between the treatment modalities in terms of condylar length differences (affected side vs. unaffected side). Therefore, the use of more conservative approaches of IMF with eventual occlusal splints may be considered in some cases of condylar neck fractures in adult subjects instead of open reductions.

### 4.2. Management of Mandibular Fractures in Children

The management of mandibular fractures in children is different compared to adults for several reasons. Firstly, children, compared to adults, tend to present greenstick fractures with little to no displaced bone [1,73,74]. Children have a higher osteogenic potential than adults, which allows a quick union within three weeks. Non-union and fibrous unions are rare in children. Due to these elements, imperfectly reduced fractures have a considerably potential and greater ability to heal correctly [79]. On the other hand, fixation of the mandible using a titanium or adsorbable plate has the potential to disrupt mandibular growth and tooth eruption because the pediatric mandible is a growing dynamic anatomic structure with missing and partially erupted teeth [51,80]. Moreover, the stabilization of rigid dental arch bars may be difficult on deciduous teeth for several reasons. First of all, there could be insufficient tooth anchorage because of the physiologic resorption of deciduous tooth roots and the incomplete root formation of the related permanent teeth. These teeth may be avulsed by the pressure induced by the procedure of dental wiring. Furthermore, the stability of rigid dental arch bars is further complicated by the conic form of the deciduous teeth, which makes them less retentive [81]. The majority of fractures in the pediatric population are therefore treated closely and nonoperatively as a result of these considerations [1,73,74].

#### 4.2.1. Condylar Fractures in Children

Cazzolla et al. [59] presented a case report of an 11-year-old boy with a unilateral condylar neck fracture with minimal displacement that was managed with a removable acrylic splint with well-defined contacts and different resin thicknesses between the right and left sides in order to keep the condyles in a centric relationship. While wearing the splint all day long and throughout meals, the patient also engaged in mouth opening exercises and muscle stretching. A similar approach was adopted by Tuna et al. [68] for a 10-year-old boy with a unilateral greenstick condylar fracture. The group of Zhao et al. [72] performed a retrospective study to evaluate the long-term effects (>1-year follow-up) of removable acrylic splints (24/24 h for 1–3 months) and functional exercises (>6 months) for the management of condylar fractures in children aged between 3 and 16 years old (deciduous, mixed, and permanent dentition). In particular, splints of 2 mm thickness were used for young children with high-level and displaced condylar fractures. Thicker splints (between three and four millimeters thick) were worn for longer periods of time by older children with low-level displaced fractures (3 months). All patients experienced clinically satisfactory outcomes with adequate occlusion, unimpaired function, and normal mandibular growth and development. Similar results were reported in the retrospective study by Sabbagh et al. [64] in which functional removable appliances were used.

Another retrospective study included in the present review [70] reported good long-term results with the association of a mini-screw and elastics for IMF and a 3 mm thick vacuum-formed occlusal splint for functional repositioning in children (<12 years) with displaced condylar fractures. Finally, a single non-comparative prospective study [58] reported good results with the use of fixed orthodontic appliances with acrylic splints and elastic + functional exercises in children with unilateral condylar fractures with displacement. However, the follow-up was only for 1 year, despite the growing subjects. However, the authors recommended guiding the healing of the condyles using progressively modified acrylic splints.

#### 4.2.2. Minor mandibular Body Fractures in Children

The case series of Agarwal et al. [56] presented a single case of a non-displaced condylar fracture managed with a cemented splint that showed slower healing due to repeated decementation and splint displacement. The other subjects were managed with IMF with arch bars or circummandibular wires (fractures with displacement). Although a conservative approach is preferred for children, the authors came to the conclusion that in some cases, ORIF is required for condylar fractures associated with symphysis/parasymphysis fractures in order to stabilize the symphysis/parasymphysis region and promote healing. Interestingly, Aizendbud et al. [57] presented a 10-year follow-up case report of a 5-year-old child with a parasymphyseal fracture with minimal displacement that was managed using a cemented rigid stainless-steel modified orthodontic splint appliance. In this case, the authors recommended closed reductions in children since growth may be affected unpredictably if the mandible’s periosteal envelope is (iatrogenically) compromised. Similar recommendations were provided in the case report papers by Chiubey et al. [61] and Sabbagh et al. [64], who managed displaced symphysis/parasymphysis fractures with cemented acrylic splints. In other cases, the cemented splint-mediated reduction of the fracture was reinforced using interdental wiring with 0.5 mm stainless-steel wire between the inferior incisors (for the management of a symphyseal fracture with minimal displacement) [63]. The use of orthodontic brackets in children was presented by Xu et al. [71], who managed a bilateral condylar and symphyseal fracture of a 10-year-old boy using bonded brackets with 0.018 in Australian wire and elastics. It is interesting to observe that after 49 months of follow-up (the patient was almost 15 years old), the left condyle displayed a little head curve, indicating incomplete remodeling, while the right condyle displayed radiologic evidence of remodeling. The fracture of the mandibular symphysis had healed.

### 4.3. Limitations and Future Directions

The results of the present review reveal that orthodontists could be a point of reference in the management of some types of mandibular fractures. However, currently the studies have mainly been conducted on children and growing subjects, and the follow-up was often not sufficient for monitoring until the completion of growth. Few studies on the conservative management of mandibular fractures included adult subjects. Furthermore, most of the studies included in this review were case reports or case series, and there were some retrospective studies. Therefore, further randomized and non-randomized clinical trials with long-term follow-ups are recommended in both growing subjects and adults in order to define the treatment guidelines and the clinical indications of the conservative orthodontic approaches for the management of mandibular fractures.

## 5. Conclusions

From the clinical perspective, the orthodontic management of mandibular fractures should be differentiated in terms of the patients’ ages and the sites of the fractures.

In children affected by condylar fractures, orthodontists or pediatric dentists should be asked to use removable acrylic splints and functional exercises or intermaxillary fixation using brackets and elastics. Furthermore, in the case of minor parasymphyseal fractures, clinicians could use acrylic splints or cemented rigid splints.

In adult subjects with minor condylar fractures, the use of orthodontic brackets or mini-screws and elastics by orthodontists has been described, whereas some minor mandibular fractures with minimal displacement have been alternatively managed with cemented acrylic splints and elastics. However, most of the articles were case reports and case series, and due to the lack of a sufficient number of clinical studies, it is not yet possible to suggest the preferential use of conservative orthodontic IMF over rigid arch bars and ORIF in adults. Therefore, further clinical studies with long follow-ups in both children and adults are needed to more precisely define the clinical indications of conservative orthodontic approaches in the management of mandibular fractures based on severity, location, and age.

## Figures and Tables

**Table 1:** Records identified in the different databases.

Database	Boolean Operators	Results
PubMed	Mandibular fracture AND (direct bonded brackets OR direct bonding technique OR splint)	552
Scopus	(“Mandibular fracture” OR “intermaxillary fixation”) AND (“direct bonded brackets” OR “direct bonding technique” OR “splint”)	497
Web of Science	Mandibular fracture AND (direct bonded brackets OR direct bonding technique OR splint)	207
Total		1256

## Data Availability

Data are available from corresponding authors upon reasonable request.
